# Cancer Care Team Functioning during COVID-19: A Narrative Literature Review and Synthesis

**DOI:** 10.3390/curroncol31010022

**Published:** 2024-01-06

**Authors:** Samar Attieh, Carmen G. Loiselle

**Affiliations:** 1 Department of Experimental Medicine, Faculty of Medicine and Health Sciences, McGill University, Montreal, QC H4A 3J1, Canada; 2 Ingram School of Nursing, Faculty of Medicine and Health Sciences, McGill University, Montreal, QC H3A 2M7, Canada; carmen.g.loiselle@mcgill.ca; 3 Department of Oncology, Faculty of Medicine and Health Sciences, McGill University, Montreal, QC H4A 3T2, Canada; 4 Segal Cancer Center, CIUSSS Centre-Ouest, Montreal, QC H3T 1E2, Canada

**Keywords:** cancer, team functioning, COVID-19, pandemic

## Abstract

Amid pandemics, health care teams face unprecedented challenges, requiring significant efforts to sustain optimal functioning and navigate rapid practice changes. It is therefore crucial to identify factors affecting team functioning in these contexts. The present narrative review more specifically summarizes the literature on key elements of cancer teams’ functioning during COVID-19. The search strategy involved four main databases (i.e., Medline OVID, EMBASE, PsycINFO, and CINAHL), as well as Google Scholar, from January 2000 to September 2022. Twenty-three publications were found to be relevant. Each was read thoroughly, and its content summarized. Across publications, three key themes emerged: (1) swiftly adopting virtual technology for communication and interprofessional collaboration, (2) promoting team resilience, and (3) encouraging self-care and optimizing team support. Our findings underscore key team functioning elements to address in future pandemics. More research is needed to document the perspectives of broader-based team members (such as patients and lay carers) to inform more comprehensive evidence-based team functioning guidelines.

## 1. Introduction

Clinical guidelines and best practices recommend a well-integrated team-based approach to cancer care that brings together complementary disciplines to optimize team processes, care management, and patient outcomes [1,2]. With the rapidly evolving and demanding nature of cancer care (e.g., higher caseloads, diverse treatment options, distinct needs and preferences of patients), teams often report feeling ill-equipped to meet these demands [3]. Pandemics pose additional challenges on team functioning, including unexpected practice changes, delays in medical procedures, cancellations of treatments, and workforce shortages due to sickness [4]. Pandemics are characterized by community-level outbreaks in at least two countries within a World Health Organization (WHO) region and at least one other country in a different WHO region [5]. In the last two decades, pandemics have mainly involved respiratory viruses, including SARS (severe acute respiratory syndrome) in 2002–2003, H1N1 influenza (swine flu) in 2009–2010, the Ebola outbreak in West Africa in 2014–2016, the Zika Virus outbreak in 2015–2016, and the COVID-19 pandemic (2019–present), considered to be one of the most significant global health crises in recent history, caused by the novel coronavirus SARS-CoV-2. During these challenging times, healthcare professionals (HCPs) have often contended with rapid shifts in clinical practices while striving to provide high-quality and uninterrupted care [4,6]. The incessant stress makes it significantly harder for teams to sustain performance and optimal functioning [7]. 

In this context, it is critical to understand key elements that affect optimal team functioning during these challenging times [8,9]. The literature shows distinct definitions and measurements of team functioning [10], with team effectiveness and relational coordination as two significant indicators [11,12,13,14,15]. Team effectiveness includes role clarity, trust, communication, coordination, timely care, staff knowledge and skills, and cohesion and problem solving [11,14]. Team relational coordination is characterized by frequent, timely, accurate, and problem-solving communication and by shared goals, shared knowledge, and mutual respect [12,13]. However, there is a literature gap regarding team effectiveness and relational coordination within cancer care settings and during pandemics. Given the timeliness of this topic, we conducted a narrative review to provide a comprehensive summary of the current literature for a quick uptake. Interestingly, the terms *narrative review* and *literature review* are often used interchangeably [16]. In addition, whereas our initial intention was to review the literature that included all types of pandemics, our search only yielded COVID-19-related publications. 

## 2. Methods

This narrative review was conducted according to the guidelines of Green et al. (2006) [17].

### 2.1. Sources of Information

The search strategy involved four main databases (i.e., Medline OVID, EMBASE, PsycINFO, and CINAHL) and Google Scholar. 

### 2.2. Search Terms and Years

Keywords included team functioning, team effectiveness, team relational coordination, cancer, oncology, cancer care, health crisis, outbreak, pandemic, epidemic, and endemic. Keywords and their respective mesh words were combined with Boolean operators and modifiers (e.g., (team functioning OR team effectiveness OR team relational coordination) AND (cancer or oncology) AND (pandemic or endemic or health crisis or outbreak)). The timeline for the search was set from January 2000 to September 2022. 

### 2.3. Publication Selection Criteria

Eligible publications focused on elements linked to cancer teams’ functioning during the COVID-19 pandemic. Special publications, such as commentaries, perspectives, reports, and contributions without quantitative or qualitative data, were included if they provided an important forum for the cancer teams’ experiences during the pandemic. Publications that were not directly relevant to cancer settings or to team functioning during the pandemic were excluded. Publications written in languages other than English or French also were excluded.

### 2.4. Data Synthesis

As per Green et al. (2006) [17], the first author (S.A.) read through each retained publications and took notes. A table was created with a synopsis of the contents and notes relevant to cancer care team functioning during the pandemic. The synopses were then reviewed, and publications with similar concepts were grouped, forming three tables. Some publications were included across tables if their content was relevant to more than one concept. Next, S.A. reviewed the synopses within each table and generated themes accordingly.

## 3. Results

**Figure 1:**
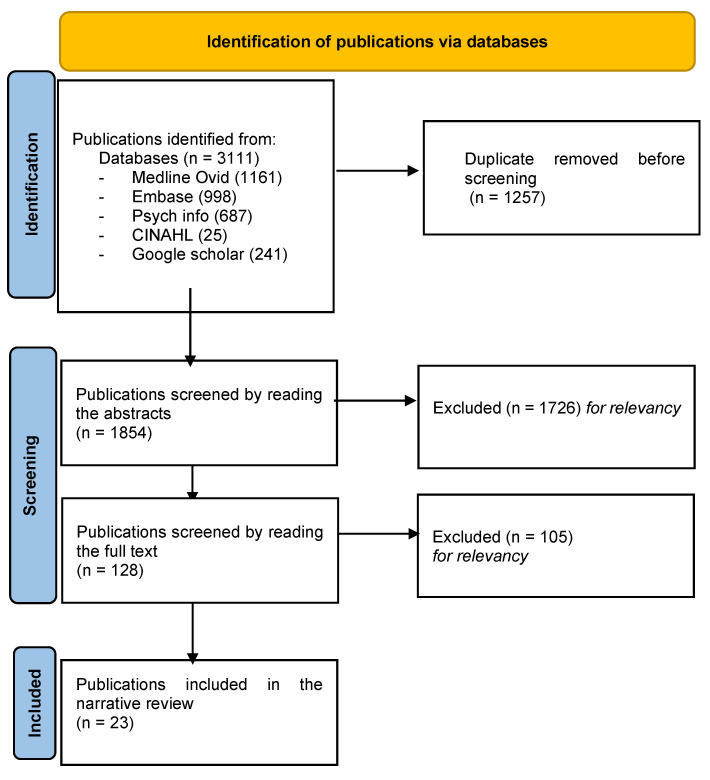
Flowchart of the publications’ selection.

A total of 3111 publications were retrieved from the databases, with the following breakdowns: Medline OVID (1161), Embase (998), PsycINFO (687), CINAHL (25), Google Scholar (241). Among these, 1257 duplicates were excluded, while 1856 were assessed for eligibility. After reading the abstracts, 1726 publications were excluded because they were not related to a pandemic. The remaining 128 were assessed by reading the full texts; 105 were not relevant to cancer care teams’ functioning or experiences during the pandemic and were excluded. The process resulted in 23 publications deemed suitable for inclusion. The PRISMA flowchart [18] in Figure 1 describes the various steps taken for identifying relevant publications.

All 23 retained publications addressed the COVID-19 pandemic. Of these, seven publications with primary sources were identified (i.e., original research with quantitative, qualitative, or mixed designs), along with one literature review, thirteen special publications (i.e., commentaries, editorial pieces, reports), and two study protocols. The main characteristics of the retained publications are presented in Table 1.

Three main themes were identified based on the in-depth review of the retained publications: (1) swiftly adopting virtual technology for communication and interprofessional collaboration, (2) promoting team resilience, and (3) encouraging self-care and optimizing team support. The publications’ synopses and associated theme(s) are presented in Table 2, Table 3 and Table 4.

**Theme** **1.** Swiftly adopting virtual technology for communication and interprofessional collaboration.

Cancer care team functioning underwent several transformations as the COVID-19 pandemic unfolded. Rapidly shifting to virtual communication and recommitting to interprofessional collaboration were evident in 13 of the publications reviewed (Table 2). 

The integration of virtual technology in cancer teams’ functioning showcased numerous advantages. Turner et al. (2022) [21] acknowledged the effectiveness of virtual technology in bringing together HCPs to coordinate care and collaborate with colleagues from within and outside institutions. Similarly, Standiford (2020) [24] emphasized the importance of high-quality virtual communication and reported that virtual technology made it possible for HCPs to collaborate with colleagues with whom they did not usually interact. Anderson et al. (2020) [29] and Soukup et al. (2021) [34] reported that switching to virtual team meetings was instrumental in improving collaborative decision-making and minimizing the impact of siloed team members on overall team performance [29,34]. Paterson et al. [25] acknowledged virtual meetings as a pragmatic and timely approach to facilitate interprofessional communication and collaboration. Ueda et al. (2020) emphasized that the rapid implementation of virtual technology during the pandemic served to maintain interprofessional collaboration, shared goals, and clear and consistent communication among HCPs. 

Farah et al. (2021) [31] reported that pre-pandemic attempts to implement virtual technology were challenging. However, during the pandemic, the urgent need for alternate methods of communication quickly became evident. Tumor boards and virtual rounds, for instance, were much easier to attend [31]. Similarly, Shah et al. (2020) [32] also reported that weekly virtual staff meetings were successful in enhancing team cohesion while providing HCPs with a sense of control amid ongoing challenges. 

Despite the benefits of implementing virtual technology, there were considerable challenges. Mohamedbhai et al. (2021) [20] and Boparai et al. (2021) [28] pointed out drawbacks such as HCPs’ struggles to adapt to various communication modalities, deteriorated communication quality, training hurdles, and reduced interprofessional engagement. Turner et al. (2022) [21] stressed the necessity for more resources to ensure virtual meetings’ quality. Moreover, Perlmutter et al. (2022) [23] highlighted the limitations of virtual meetings, particularly concerning networking with colleagues. They advocated for a hybrid model combining in-person and virtual means. Farah et al. (2020) [32] added that virtual technology can create additional clerical burden because of the lack of formalized protocols to follow. Likewise, Paterson et al. (2020) [25], Anderson et al. (2020) [29], Shah et al. (2020) [32], Soukup et al. (2021) [34], Ueda et al. (2020) [36], Jazieh et al. (2020) [38], and Reynolds et al. (2020) [40] emphasized addressing virtual technical failures and communication differences, funding IT infrastructure, and providing training for HCPs to optimize the effective utilization of virtual tools.

**Theme** **2.** Promoting team resilience.

Promoting cancer teams’ resilience to adjust to changes, overcome obstacles, and bounce back from setbacks remained central in our review, with seven publications underscoring its importance (Table 3). Banerjee et al. (2021) [19], for instance, revealed that psychological resilience and changes in working hours during the pandemic significantly predicted HCPs’ wellbeing, burnout, and job performance. In addition, 38% of HCPs (N = 1520) reported feeling burnout, and 66% were not performing their jobs effectively [19]. Le Tallec et al. (2022) [37] reported that pandemic-related constraints hindered the smooth running of oncology radiation therapists’ work, generating stress, demotivation, and loss of meaning. Similarly, Marshall et al. (2022) [22] revealed that pandemic challenges affected HCPs’ work performance and their ability to reenergize for work. Marshall et al. (2022) [22] underscored the need for HCPs’ adaptation and resilience amid significant shifts in workloads and workflows [22]. Besson et al. (2020) [30] stated that pandemics may provide an opportunity to promote team resilience if HCPs are well supported. For instance, with enough supportive resources, HCPs can grow from the experienced trauma and difficult situations [30]. 

Besson et al. (2020) [30] and Marshall et al. (2022) [22] reported that HCPs showed resilience during the pandemic, were committed to work, strived to remain unified, and did their best to cope with occupational challenges [22,30]. Besson et al. (2020) [30] added that initiatives implemented to maintain team resilience during the pandemic (e.g., weekly educational newsletters, mindfulness and resilience resources, and quizzes) enhanced team cohesion [30]. Farah et al. (2022) [31] explored strategies to build and enhance team resilience and prepare for future threats. They proposed investing in enhanced training of HCPs, hiring a robust supply of staff, enhancing virtual technologies to prevent future interruptions, and addressing burnout through tailored wellness programs and work–life balance strategies [31].

Our review also identified two study protocols on promising interventions to build and sustain team resilience in cancer settings. The first, by Tremblay et al. (2022) [26], aimed to identify contextual factors promoting cancer teams’ resilience and strategies to manage challenges post-COVID-19. The proposed intervention includes three main components aiming to monitor and prepare teams for adversity, managing their responses to challenging situations, and learning from these experiences and recovering [26]. The second protocol, developed by Chenevert et al. (2022) [27], aimed to evaluate a participatory approach that fosters team resilience, optimizes team effectiveness, and identifies critical factors linked to better organizational outcomes among cancer care teams [27].

**Theme** **3.** Encouraging self-care and optimizing team support.

During COVID-19, cancer teams faced significant stressors impacting their wellbeing and job performance. HCPs’ self-care and support for team members were critical, as outlined by ten publications (Table 4). 

Marshall et al. (2022) [22] reported that pandemic challenges significantly impacted HCPs’ wellbeing, causing higher anxiety and feelings of isolation. Banerjee et al. (2022) [28] linked HCPs’ wellbeing to their job performance and stressed the significance of supporting them to maintain high-quality cancer care. Similarly, Anderson et al. (2020) [29] highlighted the importance of supporting HCPs’ safety and wellbeing to ensure the continuity of clinical operations [22]. Davies et al. (2020) [33] reported that staff shortages and lack of resources challenge teamwork and lead to higher risk of distress among HCPs. Boparai et al. [28] revealed that pandemic-related work disruptions placed significant personal and professional demands on HCPs. Moreover, they were not able to seek informal support or engage in traditional self-care activities (such as meeting their family and friends) due to public health restrictions. In response to these challenges, Hlubocky et al. (2021) [6] underscored the responsibility of cancer organizations to support their team members. They highlighted how allocating organizational resources to tackle COVID-19-related stressors empowers HCPs for better long-term coping [6]. Hlubocky et al. (2021) [6] suggested several supportive initiatives such as ongoing needs assessment, peer or grief support groups, wellbeing support groups, mental health hotlines, and timely access to mental health specialists. Davies et al. (2020) [33] and Ngoi et al. (2020) [35] suggested boosting HCPs’ morale by establishing wellbeing hubs, mindfulness meditation videos, and weekly newsletters of available supportive resources [25,33,35].

Rosa et al. (2022) [39] argued that evidence-based interventions such as meaning-centered psychotherapy (MCP) can be adapted to promote HCPs’ wellbeing and address pandemic-related distress. MCP can facilitate team connectedness through HCPs’ openness and shared experiences [39]. Farah et al. (2021) [31] suggested several interventions for team support (e.g., wellness programs, vacations during outbreaks, reducing the stigma associated with mental health, etc.) and the creation of a chief wellness officer position to oversee supportive strategies and allocate funds [31]. Marshall et al. (2022) [22] emphasized how organizations may tend to prioritize financial aspects of the pandemic over staff safety and wellbeing. Self-care activities are therefore essential to reduce personal and professional stressors [22]. Marshall et al. added that HCPs should be watchful for the symptoms of distress among colleagues and prioritize self-care by taking breaks to reenergize and refocus [22].

## 4. Discussion

To the best of our knowledge, this narrative review is the first to provide a summary of the current literature on cancer care teams’ functioning during the COVID-19 pandemic. The publications’ findings converged toward three main themes, related to (1) swiftly adopting virtual technology for communication and interprofessional collaboration, (2) promoting team resilience, and (3) encouraging self-care and optimizing team support.

For more than two decades, a growing body of evidence has demonstrated that the care provided through interprofessional collaboration—defined as “active and ongoing partnership between professionals from diverse backgrounds working together to provide services for the benefit of healthcare users”—results in better patient outcomes [42,43,44,45]. During the COVID-19 pandemic, the complexity of sustaining in-person interprofessional collaboration fast-tracked the implementation of innovative virtual communication tools [46]. Our review revealed that virtual technology facilitated a resurgence of interprofessional collaborations among HCPs, granting them the opportunity also to engage with colleagues with whom they typically would not interact, whether within or outside their institution. Virtual team meetings have now become an integral part of cancer care, significantly changing the ways teams function and HCPs interact with one another. Virtual team communication is also documented in the broader healthcare literature. Marlow et al. (2017) [47] argued that virtual communication offers a chance for team members to learn how to use different team processes, such as coordination, to include others’ ideas and boost overall team performance [47]. They also highlighted the significant link between team performance and the timeliness and quality of virtual communication—both crucial factors during pandemics, as seen in the works of Standiford (2020) and Paterson et al. (2020) [24,25,47].

Our observations herein reveal that strong IT infrastructures and effective meeting coordination are two key strategies to enhance cancer teams’ quality of virtual meetings. This aligns with the findings of Rajasekaran et al. (2021) [48], who compared virtual meetings during the pandemic to in-person interactions. They found that, with a robust infrastructure, virtual team meetings facilitated interprofessional collaboration, both within and outside the same institution [48]. Paul et al. (2016) [49] also indicated that effective coordination of virtual teams can create positive feedback loops with trust and cohesion, improving overall team performance. 

This narrative review’s findings also indicate that further training of HCPs regarding virtual technology is needed. Particularly in the context of busy cancer care settings, the IT learning curve might be perceived as overwhelming. Being IT proficient can contribute to more effective management of work demands, as this is becoming an inherent component of healthcare practices in most settings [50]. As seen in our review, the integration of new training programs within HCPs’ curricula can help address gaps in virtual proficiencies. Kanavos et al. (2022) [51] highlighted the challenge of potential reluctance among HCPs to learn and adopt IT tools, due to a resistant mindset. Offering incentives could effectively address these issues [51].

In addition, this narrative review’s findings underscore the importance of promoting team resilience during a pandemic. Resilience, defined as “the capacity to withstand and overcome stressors that can endanger team cohesiveness and performance”, can manifest at the individual, team, and organizational levels [52,53,54]. In the context of pandemics, cancer teams need to respond collectively and adjust to work challenges in unity. Understanding the factors that underlie effective collective responses to adversity can help reveal key elements for sustained team functioning during pandemics [54]. Existing evidence, for instance, links team resilience to self-care and team support [55]. This corresponds closely to our third theme, emphasizing the importance of self-care and team support during the pandemic. Indeed, interventions to support HCPs are therefore critical to optimize team functioning when cancer teams face disruptions in routine work and higher rates of burnout [56,57,58]. Creating a supportive work environment, where team members can openly express challenges and access support, fosters team resilience [30,31,59].A survey conducted at the onset of the pandemic and three months later indicated a significant increase in the percentages of HCPs reporting distress and burnout at 3 months [19]. These findings suggest that although HCPs may be adapting effectively to change, they continue to be at increasing risk for distress [56]. Long-term supportive strategies are therefore crucial so that HCPs cope more effectively as the pandemic evolves. 

Supportive strategies summarized in this review focus primarily on HCPs, with no mention of auxiliary staff. Gasper et al.(2020) posited that such strategies should be inclusive of all cancer care team members, clinical and non-clinical (e.g., clerical and volunteers) alike [60]. As such, preparing healthcare systems to meet pandemic-related demands means addressing the entire team’s needs. However, there is a lack of evidence on the experiences of all team members [60]. According to Hlubocky (2022) [61], burnout tends to be contagious within teams [61]. When one team member experiences occupational stress, significant demands are placed on others, who, in turn, are at greater risk of developing burnout in the future [61]. Consequently, we must promptly identify and implement supportive strategies for all involved.

This review also points to gaps in our understanding of the cognitive processes affecting team functioning amid pandemics. Team members, however, have the capacity to construct mental models pertaining to their work and cultivate a collective comprehension of operational processes [14]. This is both significant and complex. Exploring team members’ mental models of team functioning can add to our shared understanding of what constitutes optimal team performance. Consequently, this can lead to the co-creation of targeted interventions and the fostering of environments that are conducive to team performance.

Despite meaningful contributions of this review, findings should be interpreted with some caution, considering the heterogeneity of publications and the sole focus on cancer care teams. The exclusion of publications addressing specific units (e.g., emergency, COVID-19 units) may have narrowed our understanding of team functioning during the pandemic. In addition, the paucity of robust studies on the topic means that we do not have much evidence on significant predictors of team functioning (positive and negative) during a public health crisis. Last, whereas our search ended in October 2022, COVID-19 continued to evolve, with relevant data still being published. 

## 5. Conclusions

This narrative review provides a comprehensive account of the literature on key elements of cancer teams’ functioning during the COVID-19 pandemic including virtual collaboration and communication, team resilience, self-care, and team support. More research is needed to document the perspectives of broader-based team members (such as patients and lay carers). The findings summarized herein can serve to inform priority domains during a pandemic so that timely strategies can be co-created among all team members involved. 

## Figures and Tables

**Table 1:** Publication characteristics (N = 23).

Author/Year	Type	Objective(s)	Participants
Banerjee et al., 2021 [19]	Original	To investigate wellbeing, burnout, and job performance in oncology over time since COVID-19	N = 1520 oncology professionals
Mohamedbhai et al., 2021 [20]	Original	To evaluate the effects of virtual head-and-neck multidisciplinary team meetings on the functioning of the team	N = 97 head-and-neck cancer team members (i.e., oncologists, nurses, pathologists, radiologists, dieticians, speech and language therapists)
Turner et al., 2022 [21]	Original	To explore oncology healthcare professionals’ experiences with the implementation of telehealth during the COVID-19 pandemic	N = 40 healthcare professionals in oncology (physicians, advanced practice providers, social workers, psychologists, dieticians, pharmacists)
Marshall et al., 2022 [22]	Original	To explore the impact of COVID-19 on oncology healthcare professionals	N = 30 (registered and advanced practice nurses, oncologists, pharmacists, mental health counselors, genetic counselors)
Perlmutter et al., 2022 [23]	Original	To identify key contributors to success and common pitfalls associated with virtual multidisciplinary meetings	N = 253 (surgeons, nurses, advanced practice providers, residents, fellows)
Hlubocky et al., 2021 [6]	Original	To describe the occupational and personal consequences of the COVID-19 pandemic on oncologists’ wellbeing and patient care	N = 25 oncologists
Standiford, 2020 [24]	Original	To explore the attributes and techniques that are important to effectively lead teams during a crisis	N= 16 physicians
Paterson et al., 2020 [25]	Literature review	To explore the role of telehealth during the COVID-19 pandemic across the interdisciplinary cancer care team	N/A
Tremblay et al., 2022 [26]	Protocol	To better understand how a multicomponent intervention builds resilience in oncology teams	
Chenevert et al., 2022 [27]	Protocol	To evaluate a participatory interventional approach that fosters team resilience and determine whether enhanced resilience improves teams’ mental health status and organizational outcomes	
Boparai et al., 2021 [28]	Special/knowledge exchange article	To discuss how oncology social workers in Australia adapted to the challenges of providing support to patients with cancer during the COVID-19 pandemic	Social workers
Anderson et al., 2020 [29]	Special/commentary	To describe the experiences of a cancer center with radiation therapy services during initial stages of the COVID-19 pandemic	N/A
Besson et al., 2020 [30]	Special/commentary	To examine the rationale for and methods of adapting a robust continuing professional development program and training for radiation therapists	N/A
Farah et al., 2021 [31]	Special/report	To identify stakeholders’ views on strategies to build healthcare resilience for future health threats	Patients, oncologists, researchers, and healthcare system representatives
Shah et al., 2020 [32]	Special/report	To highlight the transformation undertaken in a busy oncology care department to prepare for the COVID-19 crisis	N/A
Davies et al., 2020 [33]	Special/clinical correspondence	To discuss measures in place to support oncology staff throughout the COVID-19 pandemic	Oncology team members
Soukup et al., 2021 [34]	Special/editorial piece	To present lessons learned from a collaborative cancer group during COVID-19	N/A
Ngoi et al., 2020 [35]	Special/editorial piece	To present a segregated team model to maintain cancer care during COVID-19	N/A
Ueda et al., 2020 [36]	Special/special feature	To highlight the importance of organizational structure, preparation, agility, and a shared vision amidst the global pandemic	N/A
Tallec et al., 2022 [37]	Special/brief communication	To highlight teams’ and radiation therapists’ needs in the event of crisis	N/A
Jazieh et al., 2020 [38]	Special article	To present recommendations that may improve our understanding of COVID-19′s effects on cancer care and increase readiness to manage future outbreaks effectively	N/A
Rosa et al., 2022 [39]	Special/essay	To describe principles underlying a meaning-centered team-level intervention to reduce burnout among health care professionals during a health crisis	N/A
Reynolds et al., 2020 [40]	Special/perspectives	To describe teamwork and resilience in a hematology/oncology department treating patients with COVID-19	Oncology team members

**Table 2:** Team functioning synopses forming Theme 1.

Author/Year	Theme 1: Swiftly Adopting Virtual Technology for Communication and Interprofessional Collaboration
Mohamedbhai et al., 2021 [20]	58.8% believed that HCPs’ communication during virtual meetings was worse than in person 69.1% believed that interpersonal relationships and teamwork had deteriorated since moving to virtual meetings 43.9% felt that interprofessional engagement had decreased 47.7% reported that virtual training was worse than in person 70% (junior trainees) felt that their training had deteriorated since transitioning to virtual training Solutions needed to address the deficiencies in engagement, training, teamwork, and communication
Turner et al., 2022 [21]	Easier to coordinate care with other HCPs virtually Virtual technology: collaboration within the same institution and beyond institutions More resources needed to ensure the consistency and professional conduct of meetings
Perlmutter et al., 2022 [23]	Virtual board meetings: lack of opportunity to network with colleagues; connectivity issues Hybrid model to address the challenges of virtual meetings (i.e., combining virtual meetings with an in-person component) Meetings’ leadership reinforces engaged interprofessional participation
Standiford, 2020 [41]	The pandemic created unique circumstances for team collaboration/made high-quality communication a necessity The pandemic challenged typical interprofessional communication practices, with HCPs overwhelmed by electronic communication Opportunity to work in multidisciplinary teams and learn from colleagues (not normally interacted with pre-pandemic)
Paterson et al., 2020 [25]	Telehealth and virtual technology/more effective and sustainable models of care Benefits and limitations of virtual technology need careful consideration Need for appropriate HCP training and education Virtual multidisciplinary cancer team meetings/a pragmatic interprofessional approach, timely and safe More engagement from allied professionals possible virtually/providing high-quality advice to teams Telehealth’s functionality brings together expert clinicians and carers (even if geographically dispersed) and facilitates interprofessional collaboration, which is known to improve clinical performance, patient outcomes, and patient satisfaction HCP curriculum development needed in undergraduate and postgraduate studies
Boparai et al., 2021 [28]	Ongoing change in communication forums during the pandemic HCPs challenged in understanding and implementing large amounts of information Challenge of adaptation to different communication modes
Anderson et al., 2020 [29]	Creation of “siloed” teams significantly impacted traditional forms of communication Workforce communication strategy: replace face-to-face with virtual Regular interactive staff briefings to communicate planning and decisions Information technology infrastructure Staff education to support remote access to all resources
Farah et al., 2021 [31]	Pre-pandemic attempts to implement virtual technology were challenging During the pandemic, HCPs committed to rapid changes in communication, work, and collaboration when faced with urgency Rapid transition to virtual care Benefits of virtual technologies: relieve some space/time constraints; tumor boards easier to attend Challenges of virtual technologies: clerical burden; less interaction with colleagues
Shah et al., 2020 [32]	Changes in operating procedures pertaining to communication Development of a regular line of communication among division leaders through video conferencing Daily leadership video conference call between professionals HCPs’ adaptations centered around communication and coordination within teams and externally Benefits of virtual meetings: cohesive team/HCPs with a sense of control
Soukup et al., 2021 [34]	Videoconferencing improved collaborative decision-making Challenge to quality decision-making: technology failure and differences in communication styles Hybrid model: supplement virtual with periodic face-to-face interaction/more nuanced communication
Ngoi et al., 2020 [35]	Clear communication recognized as being key to minimizing uncertainty among HCPs Rapid communication ensured quick implementation of protocols and changes Teleconferencing was utilized for interprofessional meetings and HCPs’ education
Ueda et al., 2020 [36]	Necessary to centralize information, to consolidate and communicate the work Framework for interprofessional collaboration toward a shared goal Rapidly expanded telemedicine efforts through expedited physician credentialing, training, and modification based on changing regulations Enabling work-from-home by prioritizing information technology resources Virtual meetings are essential for clear and consistent messaging
Jazieh et al., 2020 [38]	Timely virtual access was needed to facilitate the exchange of information and concerns between staff and organizational administration/leaders Organizations need to invest in appropriate infrastructure (i.e., adequate hardware and internet bandwidth) Training needed on the optimal use of telehealth and how to effectively communicate on a virtual platform Strong communication between HCPs is critical to ensure well-coordinated work Challenges of virtual technology need to be addressed
Reynolds et al., 2020 [40]	Transition to virtual technology had a big impact on cancer team s Quick adaption to ever-changing recommendations, communication, and working conditions Importance of ascertaining the needs of each HCP Training HCPs and planning for additional staffing to meet the demand

**Table 3:** Team functioning synopses forming Theme 2.

Author/Year	Theme 2: Promoting Team Resilience
Banerjee et al., 2021 [19]	38% of HCPs (N = 1520) reported burnout; 66% did not feel that they were performing their job effectively Psychological resilience and work hours predicted wellbeing and burnout
Marshall et al., 2022 [22]	Lower ability of HCPs to rejuvenate and reenergize for work Limited resources; concerns for the mental health of HCPs; need for adaptation Resilience amid shifting workloads, workflow, and new restrictions
Tremblay et al., 2022 [26]	Mechanisms promoting team resilience, courses of action in difficult situations, mechanisms for problem resolution, and realistic solutions to professional workforce and team effectiveness challenges. The BRIOT intervention: monitoring and preparing for situations of adversity (minimizing), coping with responses to adversity (managing), and recovering and learning from the experiences (mending)
Chenevert et al., 2022 [27]	Resilience = core component of effective multidisciplinary team functioning Integrative organizational model of resilience Recommended interventions to increase individual and team resilience
Besson et al., 2020 [30]	Promoting resilience and acceptance of change Supporting HCPs to foster growth from trauma and/or stressful circumstances Staff striving to remain unified and connected Educational and social support focusing on the long-term morale of teams Strategies to maintain resilience: weekly educational newsletter with mindfulness and resilience sections, a quiz, and reflection sections Team cohesion as a positive effect of the implemented strategies
Farah et al., 2021 [31]	Canadian healthcare system contributions to resilience regarding future threats Strength-related concepts as a springboard to building resilience Recommendations: invest in wellness programs, chief wellness officers, and funds allocated for support
Le Tallec et al., 2022 [37]	Constraints that hinder the smooth running of planned programs, generating stress, demotivation, and loss of meaning in the exercise of the profession (ethical challenges)
Hlubocky et al., 2021 [6]	Leadership supporting resilience Interventions to build a supportive, ethical work climate to restore resilience Optimal evidence-based programmatic interventions

**Table 4:** Team functioning synopses forming Theme 3.

Author/Year	Theme 3: Encouraging Self-Care and Optimizing Team Support
Banerjee et al., 2021 [19]	Burnout significantly associated with poorer wellbeing Wellbeing support services accessible to participants Combination of supportive approaches (e.g., online or smartphone apps, psychological support from work, and telephone support) Coping strategies (e.g., thinking of positives, changes in physical activity, talking to colleagues to get information, and using humor or laughing)
Marshall et al., 2022 [22]	Feelings of isolation and expressions of mental difficulties and challenges Further feelings of isolation caused by distance from loved ones/higher anxiety Recommendations needed for self-care activities and stress management Self-care should be addressed during working hours (e.g., taking breaks throughout one’s shift to reenergize and refocus) HCPs should be watchful for psychological distress symptoms among coworkers Importance of self-care to reduce personal/professional stressors Organizations not putting the safety of the employees first/concerned about the financial aspect
Boparai et al., 2021 [28]	Work has been demanding, professionally and personally Consideration of HCPs’ wellbeing must remain a focus Challenge: HCPs’ face-to-face informal peer support opportunities not permitted Challenge: established self-care techniques (e.g., meeting family and friends) restricted
Anderson et al., 2020 [29]	Minimizing the risk of exposure to COVID-19, for the health and wellbeing of the HCPs and the continuity of clinical operations The Compassion and Resilience Education (CARE) program/peer support Wellbeing initiatives/mindfulness meditation videos /tips on home isolation Integration of department-supported “socially distanced” morning teas for siloed staff and virtual “after work drinks” in an attempt to boost staff morale Encouraging leave to continue to be taken (where possible), to ensure a mental break Regular “check-ins” with staff to ensure that their needs are being met, that they are well and safe, and that any barriers to their work are being addressed
Farah et al., 2021 [31]	Support strategies (e.g., investing in wellness programs; creating a healthy environment where workers are not overworked and can relax, meditate, or simply sit in silence; structured multidisciplinary teams for psychosocial support; allowing for planned vacations even during an outbreak; implementing strategies to reduce the stigma associated with mental illness; allowing flexibility in work shifts; providing mental health support; investing in wellness programs; creating positions such as chief wellness officers responsible for creating support and allocating funds to support)
Davies et al., 2020 [33]	Pandemic demands; staff vulnerability to moral injury Staff shortages and lack of resources can result in challenging ways of working, leading to psychological distress Strategies (e.g., access to psychologists; coping effectively; information and practical resources; evidence-based online workshop for the prevention of post-traumatic stress disorder; collaboration with the cancer psychology service; weekly bulletin to easily access resources and helpful strategies) Available resources monitored and revised to meet staff needs Collaboration between the lead cancer nurses and the cancer psychology service to meet the needs of oncology staff
Ngoi et al., 2020 [35]	Staff morale expected to be affected by the workload of team segregation, cancellation of leaves, and enforced social distancing Strategies to boost morale (e.g., sharing of appreciation messages, provision of refreshments, and the setup of a group chat to share anecdotes, information, and banter)
Ueda et al., 2020 [36]	Reassignment of clinical duties to administrative roles (i.e., HCPs who are immunocompromised or have significant comorbidities/increased risk from COVID-19). Emotional and physical wellbeing of HCPs requires proactive attention HCP burnout is expected; importance of self-care/downtime for rest
Rosa et al., 2022 [39]	Anxiety, helplessness, experiences of grief and loss; conflicting emotions during the pandemic Amplification of burnout in HCPs; disconnection from the sense of meaning and purpose Self-care is a triggering concept Potential power of MCP to facilitate team connectedness and meaning-making Engaging in MCP; sharing of mutual points of existential distress; fostering a connectedness between the HCPs and an opening to come together
Hlubocky et al., 2021 [6]	HCPs at risk of moral strain, in the form of moral distress and moral injury Strategies for institutional wellbeing programs (i.e., assessment of oncologists’ needs, proactive engagement of leadership and mental health in collaborative action planning, establishment of oncology wellbeing programs, execution of empirical-based wellbeing interventions, reassessment of needs, and modification of interventions as needs change). Organizational resource investment in addressing COVID-19-related stressors/empowering HCPs for long-term coping Promoting and supporting wellbeing and professional fulfillment at the organizational and individual levels
